# Potential role of intestinal microflora in disease progression among patients with different stages of Hepatitis B

**DOI:** 10.1186/s13099-020-00391-4

**Published:** 2020-10-27

**Authors:** Xiu-An Yang, Fengchun Lv, Ran Wang, Yange Chang, Yiming Zhao, Xinyu Cui, Haochen Li, Sixi Yang, Suting Li, Xuemin Zhao, Zhishuo Mo, Fang Yang

**Affiliations:** 1 School of Basic Medical Science, Chengde Medical University, Anyuan Road, Chengde, 067000 People’s Republic of China; 2 Department of Pharmacology, Chengde Medical University, Chengde, 067000 People’s Republic of China; 3 State Key Laboratory of Quality Research in Chinese Medicine and School of Pharmacy, Macau University of Science and Technology, Macau, People’s Republic of China; 4 Clinical Skills Center, Chengde Medical University, Chengde, 067000 People’s Republic of China; 5 Department of Infectious Diseases, The Third Affiliated Hospital of Sun Yat-Sen University, No 600, Tianhe Road, Guangzhou, 510620 People’s Republic of China; 6 Institute of Biophysics, Chinese Academy of Sciences, 15 Datun Road, Beijing, 100101 People’s Republic of China

**Keywords:** Gut microbiota, Disease progression, Hepatitis B, 16S rRNA gene sequencing, Cirrhosis, Acute-on-chronic liver failure

## Abstract

**Background:**

Increasing evidence demonstrate that the gut microbiota is involved in the pathogenesis of liver diseases, and faecal microbiota transplantation is considered to be a promising new treatment option. However, there are no reports on the intestinal flora of asymptomatic HBV carriers using next-generation sequencing. This study intends to investigate the potential role of the intestinal microflora in predicting the progression of Hepatitis B patients in different non-cancerous stages.

**Results:**

A total of 266 patients with different stages of Hepatitis B and 31 healthy controls were included in this study. Some of the subjects (217 cases) underwent 16S rRNA gene sequencing. Compared with the control group (CK), the α diversity of patients in Group A (HBV carrier) slightly increased, while that of patients in the other three groups decreased. Each group of patients, especially those in Group C (cirrhosis) and Group D (acute-on-chronic liver failure), could be separated from the CK using weighted UniFrac PCoA and ANOSIM. LEfSe revealed that 40 taxa belonging to three phyla had an LDA larger than 4. In addition to the comparison between Group B (chronic Hepatitis B) and Group C, the specific flora and potential taxonomic function were also identified. Different microbial communities were found to be highly correlated with clinical indicators and the Child-Pugh scores. Changes in the microbial community were highly related to the alternations of host metabolism, which in turn, was related to the development of Hepatitis B. Our analysis identified a total of 47 strains with potential biomarker functions at all levels except for the phylum level.

**Conclusions:**

Faecal microbiota transplantation of some potential beneficial bacteria can change with the occurrence of disease, and HBV carriers might be the most suitable donors.

## Background

Hepatitis B is caused by hepatitis B virus (HBV) infection and can lead to acute and chronic liver diseases [1]. Patients with chronic Hepatitis B (CHB) often develop serious complications, such as cirrhosis and hepatocellular carcinoma (HCC). HCC is the third leading cause of cancer death worldwide [2]. At present, safe and effective vaccines can prevent Hepatitis B [3]. However, according to the WHO, there were as many as 1.1 million new cases of Hepatitis B in 2017 (https://www.who.int/news-room/fact-sheets/detail/hepatitis-b). In China, 80% of all HCC diagnoses are attributed to Hepatitis B [3]. Even in the USA, the incidence rate of HCC has increased rapidly [4]. Therefore, Hepatitis B is a global public health problem threatening human health.

As of 2016, only 10.5% (27 million) of all estimated Hepatitis B patients knew that they were infected, and 4.5 million (16.7%) confirmed patients were receiving treatment (https://www.who.int/news-room/fact-sheets/detail/hepatitis-b). HCC progresses rapidly; most patients are usually diagnosed at an advanced stage, and the prognosis of advanced liver disease is generally poor [5]. Therefore, early prediction of Hepatitis B progression is of great value for the diagnosis and treatment of HCC.

Faecal microbiota transplantation (FMT) is considered to be a promising new treatment option for a variety of refractory diseases, including Hepatitis B [6, 7]. Increasing evidence demonstrates that the gut microbiota is included in the pathogenesis of liver diseases [8–10]. At present, studies on the intestinal flora in patients with Hepatitis B have mainly focused on patients with CHB or patients with advanced liver diseases caused by liver cirrhosis or HCC [8–12]. As far as we know, there are no reports on the intestinal flora of asymptomatic HBV carriers using next-generation sequencing. Therefore, a cross-sectional study including Hepatitis B patients at different stages was conducted. We aim to have a comprehensive understanding of the intestinal flora of patients with hepatitis B by studying all non-cancerous hepatitis B patients.

## Results

### Clinical characteristics of the cohort

After filtration, 297 subjects were included in this study. Among the included participants, 217 had undergone 16S rRNA gene sequencing. In this section, 31 cases were healthy controls (Control or CK), 24 cases were asymptomatic HBV carriers (Group A), 56 cases had CHB (Group B), 54 cases had liver cirrhosis (Group C), and 52 cases had acute-on-chronic liver failure (Group D). The remaining 80 cases only underwent clinical trials, with 20 cases in each disease group. Additional file 1: Table S1 shows the details of the clinical trials for this cohort. It is worth noting that the patients in Group C and D were obviously older than those in group A and B, indicating that age might be related to disease progression.

Discriminant analysis was performed using clinical data. The data of patients involved in 16S rRNA gene sequencing were regarded as training data. As indicated in Additional file 2: Table S2, seven indexes were included in the discriminatory function. The classification accuracy of the initial grouping and cross-validation cases were 85.1% and 82.6%, respectively. The accuracy of the cross-validation in Groups A-D was 90.0%, 74.5%, 79.6% and 92%, respectively. An additional 80 patients were included in the validation queue, and 68 cases (85.0%) were correctly classified. Our results suggest that it is possible to divide patients with non-cancerous Hepatitis B into different stages according to clinical tests. However, the classification efficiency for patients with CHB and liver cirrhosis is somewhat poor.

### Overview of the microbiota diversity in the cohort

**Fig. 1:**
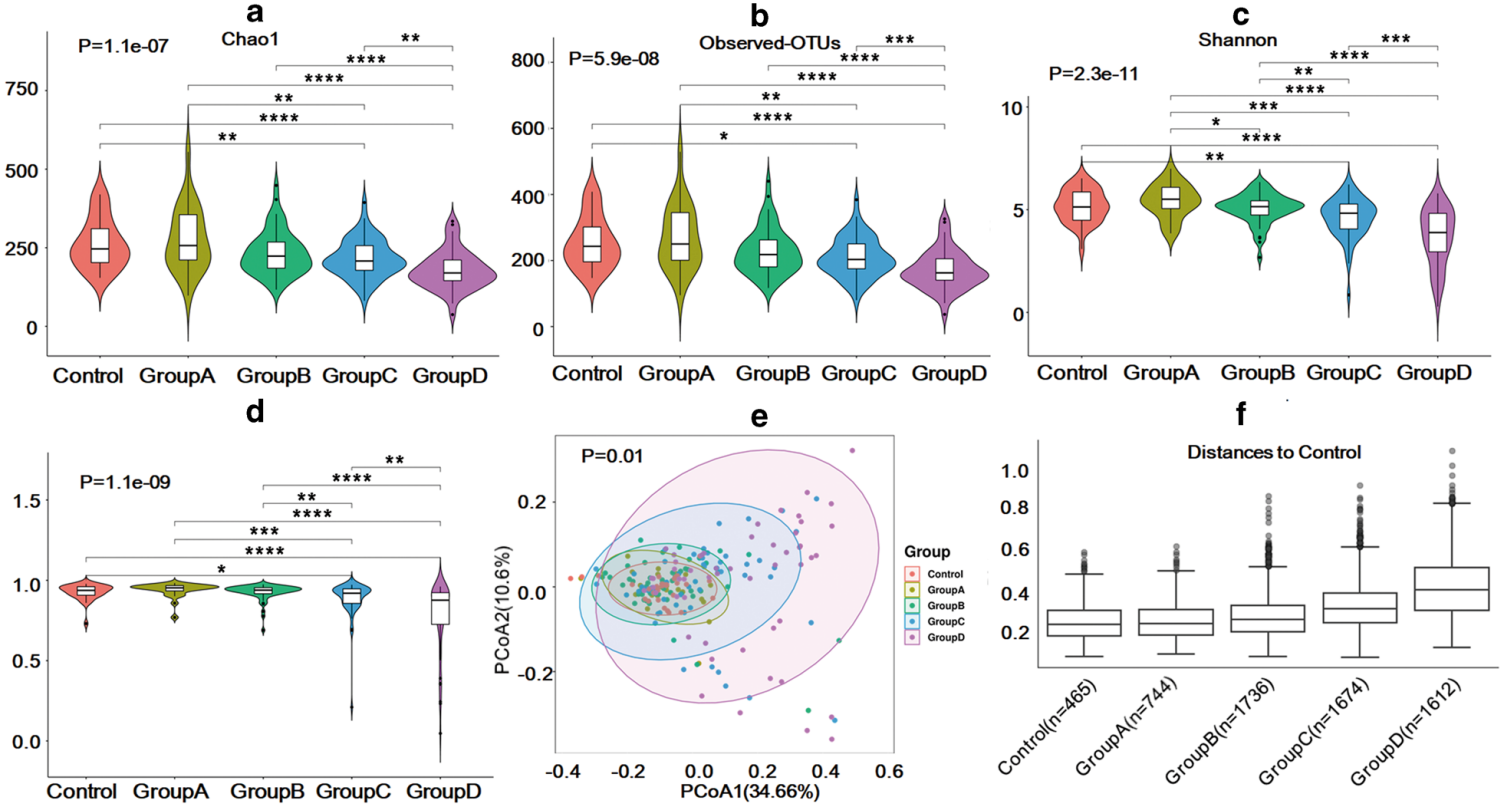
Comparison of the microbial community structure in each group. Compared with CK, the α diversity revealed by Chao1, the observed OTUs, the Shannon index, and the Simpson index of Group **a** increased slightly, while that of the other three groups decreased (**a**–**d**). Significant differences (p < 0.05) are marked with an asterisk. The more asterisks, the greater the difference. Beta-diversity, calculated using principal coordinate analysis (PCoA) and analysis of similarities (ANOSIM), showed that the microbiota of the five groups could be well differentiated (p = 0.001, **e**, **f**)

To clarify the characteristics of the intestinal microflora in different stages of Hepatitis B patients, 16S rRNA gene sequencing was performed. Wilkerson rank-sum tests showed significant differences in Chao1 (p = 1.1e^−7^), observed OTUs (p = 5.9e^−8^), the Shannon index (2.3e^−11^), and the Simpson index (1.1e^−9^) among groups. Compared with the CK, the α diversity of Group A increased slightly, while the diversity of the other three groups decreased (Fig. 1a–d, Additional file 3: Table S3). PCoA showed that there were no significant differences in age and gender (Additional file 4: Figure S1). Each group of patients could be separated from the CK by weighted UniFrac PCoA (p = 0.001, Fig. 1e) and UniFrac ANOSIM (p = 0.001, Fig. 1f). In sum, our research results showed that compared with the CK, the microbial community diversity of patients in Group C and Group D decreased significantly, and patients with Hepatitis B in different stages could be classified according to their intestinal flora.

### Alterations of the taxonomic composition in Hepatitis B patients

**Fig. 2:**
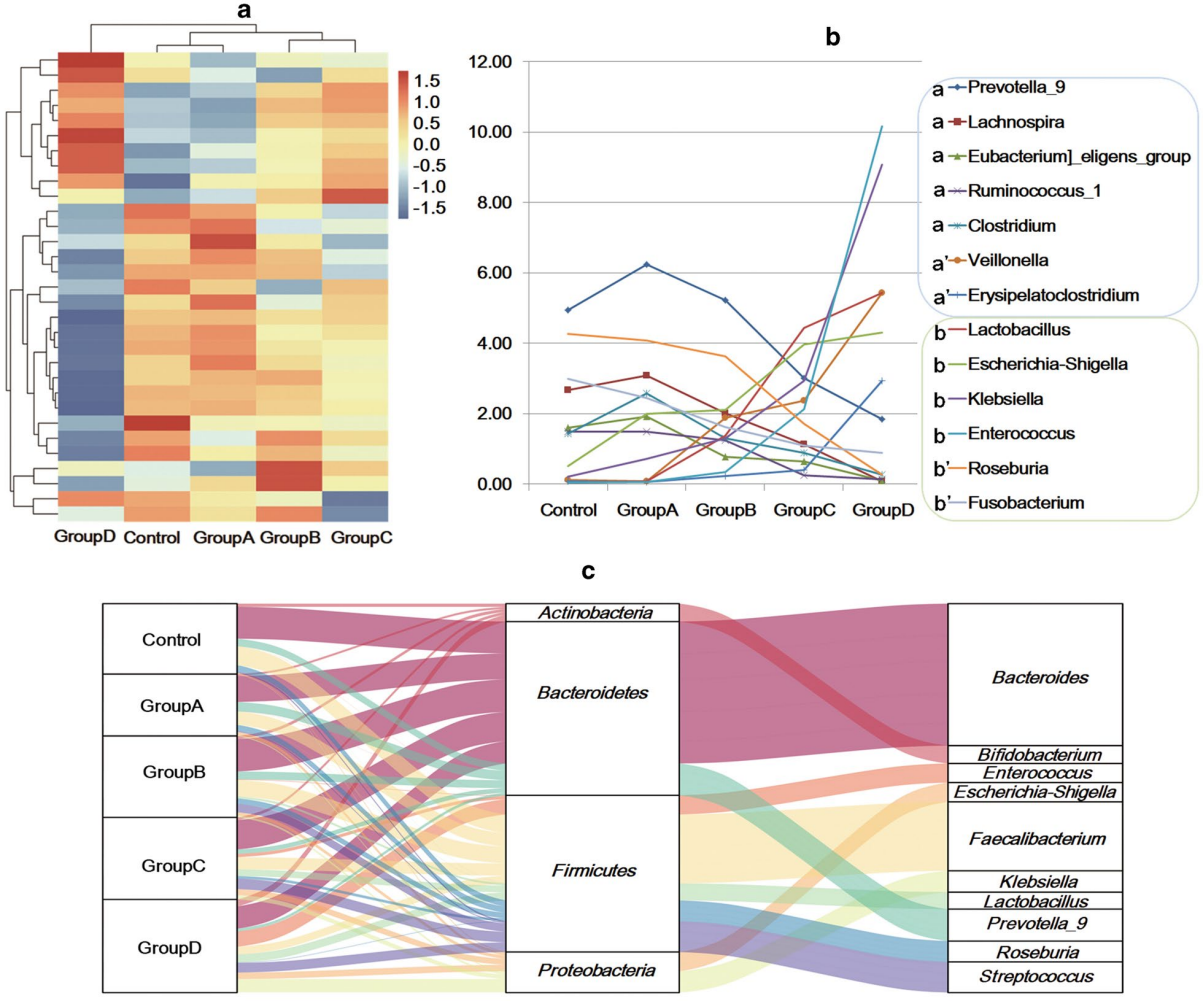
Abundance of the top top-level microflora at the phylum and genus levels. Heat map of the Top 30 genera (**a**) showed that the composition of the microbial groups changed with the progression of disease, and Group A had specificity (**b**). Bacteria within the blue frame increased in Group A and then decreased in Groups B–D (a) or decreased in Group A and then increased (a′). Bacteria within the green frame increased (b) or decreased (b′) with the development of the disease. The corresponding proportion of the floral diversity at the phylum and genus levels (**c**). Note: The strains from top to bottom were: *Bacteroides*, *Faecalibacterium*, *Streptococcus*, *Prevotella_9*, *Klebsiella*, *Enterococcus*, *Escherichia*-*Shigella*, *Lactobacillus*, *Bifidobacterium*, *Roseburia*, *Veillonella*, *Ruminococcus]_gnavus_group*, *Parabacteroides*, *Megamonas*, *Agathobacter*, *Lachnospiraceae_unclassified*, *Subdoligranulum*, *Fusobacterium*, *Lachnospira*, *Blautia*, *Clostridium*, *Ruminococcus_2*, *Ruminococcus]_torques_group*, *Sutterella*, *Erysipelatoclostridium*, *Prevotella_2*, *Eubacterium]_eligens_group*, *Lachnoclostridium*, *Ruminococcus_1*, *Collinsella*, and Others

To understand the changes of the classification components, after labelling with Silva and NT-16 databases, abundant features were detected at different levels. In all stool samples, the abundance of bacteria at the phylum, class, order, family, genus and species levels was 26, 67, 142, 249, 637, and 1154, respectively. The average abundances of microbial communities at the phylum level and the Top 30 microbial communities at genus level are shown in Additional file 5: Figure S2. The phyla Choerosclerotis, Bacteroides, Proteus, Actinomycetes and Fusobacterium accounted for more than 98% of all phyla in all groups (Additional file 5: Figure S2A and Additional file 6: Table S4). The heat map showes that the Top 30 microbial communities at the genus level generally varied at different stages (Fig. 2a). *G_Prevotella_9*, *g_Lachnospira*, *g_Eubacterium]_eligens_group*, *g_Ruminococcus_1*, and *g_Clostridium* increased in Group A and then decreased in Group B-D while *g_Veillonella* and *g_Erysipelatoclostridium* decreased in Group A and then increased in B-D (Fig. 2b Additional file 7: Table S5). The Sankey plot shows that *g_Bacteroides*, *g_Prevotella_9*, and *g_Roseburia* reduced while *g_Enterococcus*, *g_ Escherichia–Shigella*, and *g_Streptococcu* increased with disease progression (Fig. 2c and Additional file 8: Table S6). To summarize, the taxonomic composition altered with disease progression, and the gut microbiota of the patients in Group A showed specificity.

### Specific microbiological marker identification in patients with different stages of Hepatitis B

**Fig. 3:**
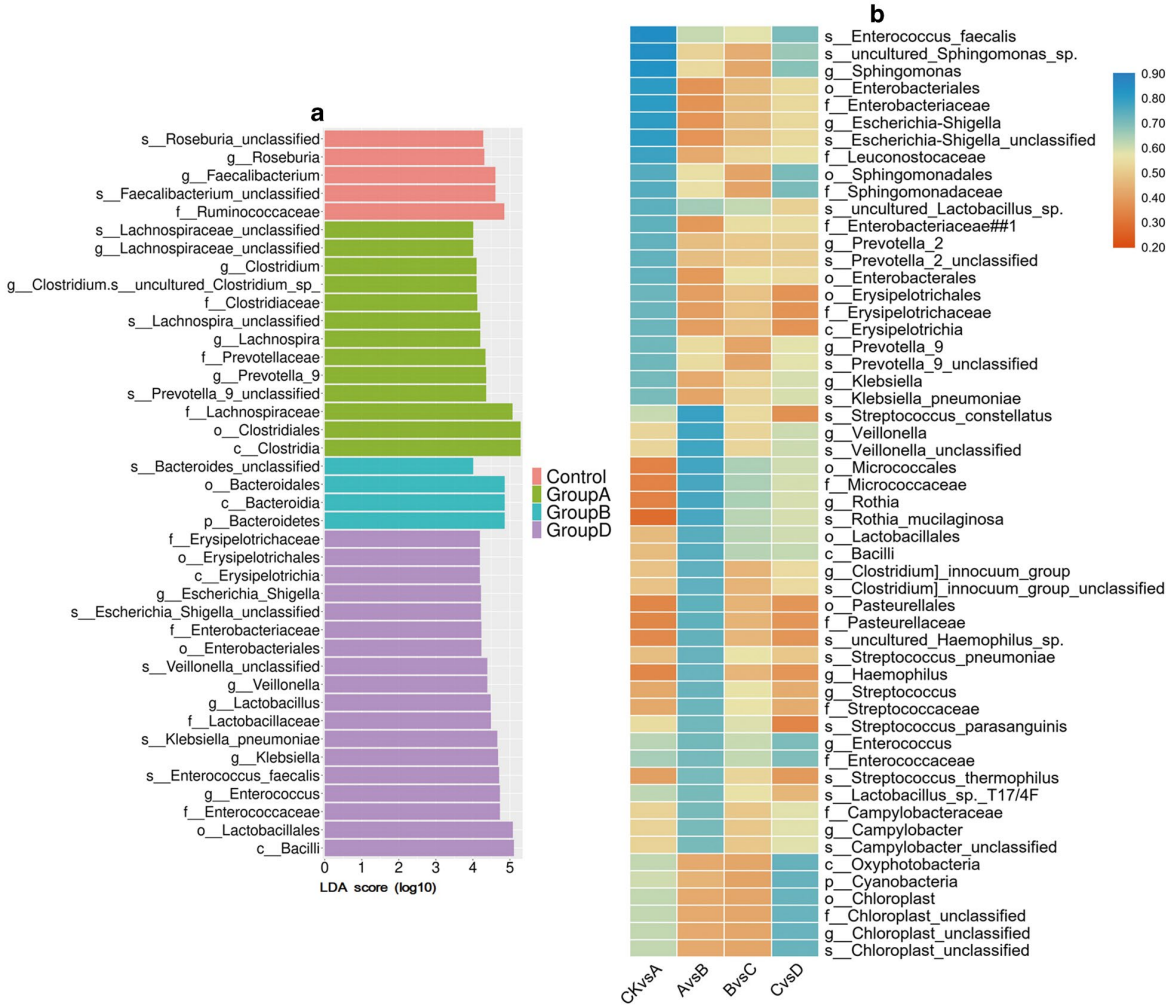
Most differentially abundant taxa in the faecal microbiota identified by LEfSe (**a** LDA > 4) and flora with an area under the curve (AUC) value of more than 0.7 (**b**). CKvsA, analysis between the control and Group A; similar hereafter

In order to investigate the specific bacterial taxonomic group related to Hepatitis B procession, the faecal microorganisms in this cohort were compared by using LEfSe. A total of 40 taxa belonging to three phyla (Bacteroides, Sclerotinia, and Proteus) had an LDA value greater than 4 (Fig. 3a and Additional file 8: Table S6). These three phyla were also the Top 3 most abundant flora. No obvious bacteria were found in Group C, but 18 specific bacterial groups were found in group D. The dominant bacteria of the CK were *g_Roseburia* and *g_Faecalibacterium*, both belonging to *p_Firmicutes*. Group A was dominated by *g_Prevotella_9*, *g_Clostridium*, *g_Lachnospira*, and *g_Lachnospiraceae_unclassified*, of which the most dominant was *p_Bacteroidetes*, while the other groups were dominated by *p_Firmicutes*. The bacteria *o_Bacteroides* had an advantage in Group B. The diversity of the dominant bacteria in group D increased, and the main strains were *g_Enterococcus*, *g_Lactobacillus*, *g_Veillonella, g_Klebsiella* and *g_Escherichia_Shigella* (Fig. 3a and Additional file 8: Table S6). The LDA values of *c_Clostridia*, *o_Clostridiales*, *c_Bacilli*, *o_Lactobacillales*, and *f_Lachnospiraceae* were greater than 5. In summary, these 40 diverse flora may be potential biomarkers for Hepatitis B patients.

The diagnostic threshold was calculated using a ROC curve to determine the significance of the microbiota sequencing results for Hepatitis B classification. As shown in Fig. 3b and Additional file 9: Table S7, in addition to the comparison between Group B and group C, a specific flora with a potential taxonomic function (AUC > 0.7) was identified. Most of the identified bacteria only appeared once, and only *s_Enterococcus_faecalis*, *o_Sphingomonadales*, *f_Sphingomonadaceae,* and *g_Enterococcus* were identified twice, which indicated that the flora of Hepatitis B patients changed greatly with the progression of the disease. Seventeen kinds of bacteria were found by LEfSe and ROC curve analysis, indicating that they might be specific microbial markers of Hepatitis B. Half of the identified 17 kinds of bacteria were found in abundance analysis at the genus level (Additional file 9: Table S7).

### Potential classification efficiency for GDI

**Fig. 4:**
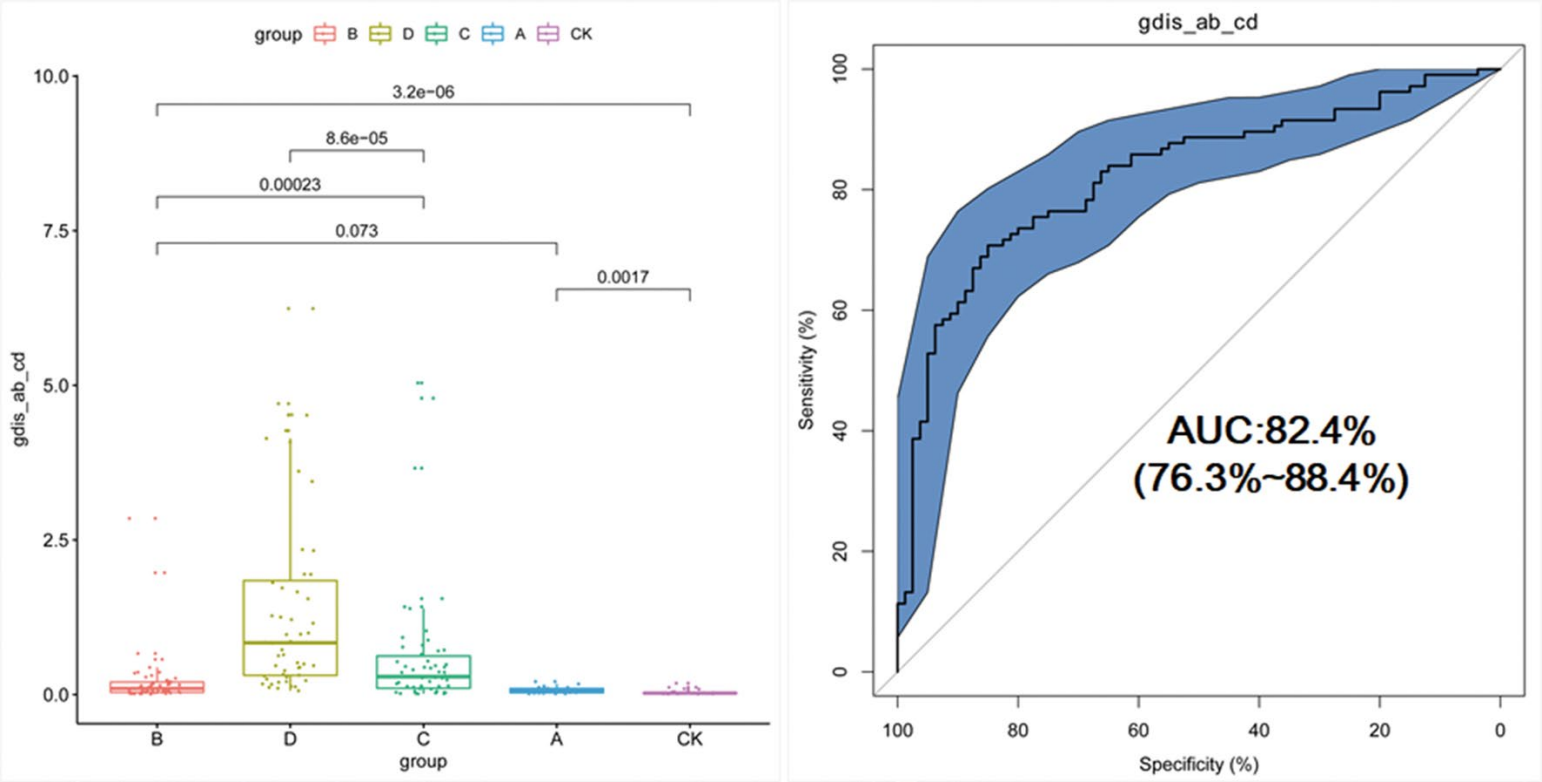
Classification of Hepatitis B patients with (Group A & B) and without (Group C & D) liver cirrhosis using the gut dysbiosis index (GDI)

Since the AUC calculated by using the single flora shared between Group B and Group C was less than 0.7, we then investigated the identification efficiency of GDI for patients with and without liver cirrhosis. The achieved AUC between the non-cirrhosis group (Group A and Group B) and the liver cirrhosis group (Group C and Group D) was 0.824, with a 95% CI of 0.763 to 0.884 (Fig. 4). This result demonstrated that the GDI based on microflora has satisfactory diagnostic potential for cirrhosis and non-cirrhosis patients.

### Relationship among faecal microorganisms, clinical indicators and the progression of Hepatitis B

**Fig. 5:**
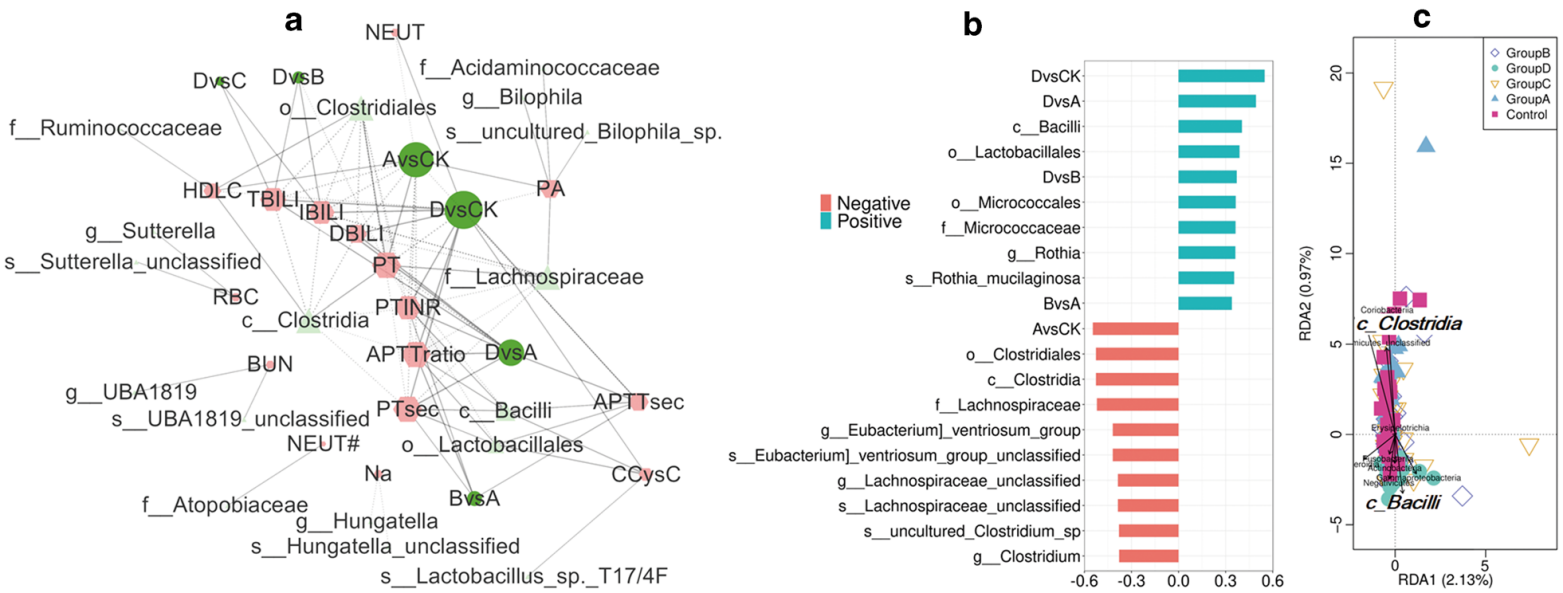
Correlation between the intestinal microflora and clinical indexes. Correlation analysis between differentially abundant taxa determined by LEfSe and the clinical indexes (**a** r > 0.4). The results are displayed with Cytoscape. Hexagon, clinical indexes; circle, GDI; triangle, bacteria. The size of the graph is determined by the number of related lines. A solid line indicates a positive correlation, and a dashed line indicates a negative correlation. The thickness of the line is determined by the correlation coefficient. Correlation analysis between differentially abundant taxa revealed by LEfSe and the Child-Pugh score (**b** r > 0.3). The association between the Top 10 genera and Hepatitis B stages revealed by redundancy analysis (RDA)

To better study the relationship between the faecal microflora and clinical indicators, the correlation among clinical indicators, the dyspepsia index and differential microflora was analysed. Strong correlations (|correlation r| > 0.4) were used for network construction, and the result was displayed with Cytoscape (v3.72, https://cytoscape.org/). As shown in Fig. 5a and Additional file 10: Table S8, *c_Bacilli* and *c_Bacilli|o_Lactobacillales* were positively correlated with coagulation function and CCysC. *C_Clostridia* was negatively correlated with coagulation function and bilirubin metabolism. These indicators accounted for most of the identified connections (Fig. 5a and Additional file 10: Table S8). It is worth noting that *g_Hungatella*, which belongs to *o_Clostridiales*, was negatively associated with blood sodium; that is, it was positively linked to disease progression. Red blood cells were positively correlated with *g_Sutterella*.

To further verify our results, the Child–Pugh score was used to calculate the severity of liver damage, and then, the correlation between liver injury and microorganism was analysed. A total of 15 flora belonging to *p_Actinobacteria* and *p_Firmicutes* were identified (Fig. 5b and Additional file 11: Table S9). Consistent with results shown in Fig. 5a, *c_Bacilli* and *c_Clostridia* were inversely correlated with the clinical information. All of the 15 identified flora overlapped with the bacteria identified by significant LDA or the AUC, which indicates that they are related to disease progression and have potential value for clinical diagnosis (Additional files 8, 9, 11: Tables S6, S7, S9). Then, redundancy analysis (RDA) was performed on each level of all the samples, and the relationship between the intestinal flora and Hepatitis B staging was revealed according to the Top 10 enriched flora. Similarly, *c_Bacilli* and *c_Clostridia* were oppositely associated with the clinical information (Fig. 5c). In conclusion, our results strongly support the theory that faecal microorganisms are related to clinical indicators and Hepatitis B progression.

### Changes of inferred microbial function

To predict the organism-level coverage of functional pathways and bio-interpretable phenotypes (such as Gram staining and oxygen tolerance) at the organism level, the intestinal microbial community was analysed by using BugBase [13]. Pairwise Mann–Whitney-Wilcoxon tests were performed, and the results are shown in Additional file 12: Figure S3. No significant difference was observed in Gram-positive and Gram-negative flora in each group compared with the CK; however, a significant difference was found in Group D compared with Group A. Aerobic stats, Contains Mobile Elements stats, Facultatively Anaerobic stats, and Stress Tolerant generally changed with disease progression.

**Fig. 6:**
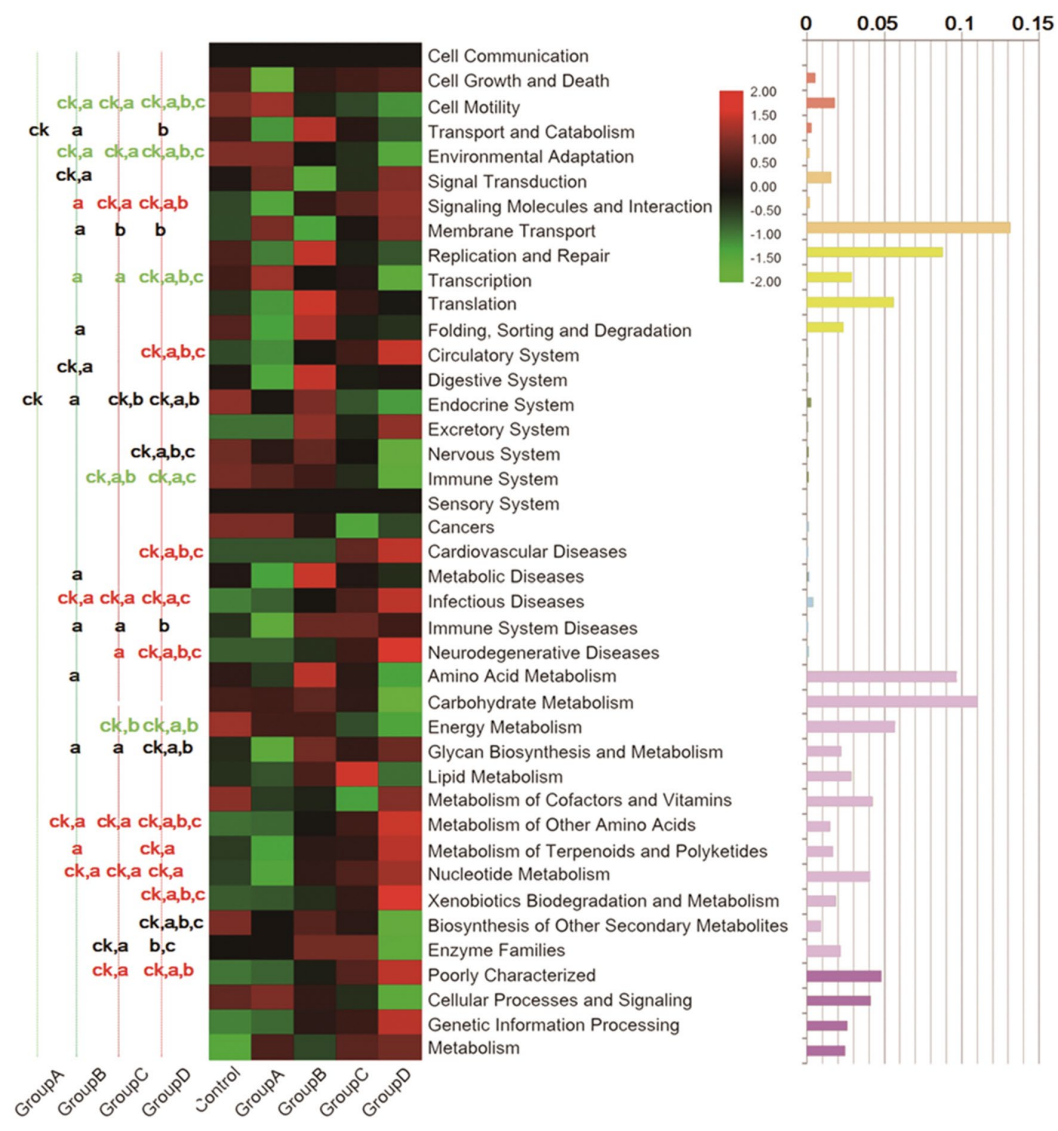
Microbial community function was predicted by PICRUst. A total of 41 biological pathways were identified and divided into 7 categories. Note: the letters CK, a, b, and c on the left panel indicate *p* < 0.05. For example, “CK” is *p* < 0.05 compared with the healthy controls

PICRUst was used to determine the changes of the predicted microbial functions in the bacterial communities [14]. We identified 41 kinds of KEGG biological functions, which could be divided into seven categories (Fig. 6). Membrane transport (13.11%), carbohydrate metabolism (10.98%), amino acid metabolism (9.64%), replication and repair (8.76%), energy metabolism (5.69%), and translation (5.58%) accounted for more than 5.0% of the functions (Additional file 13: Table S10). Significant differences were found in more than half of the identified functions. Compared with the CK, only the endocrine system and transport and catabolism of CHB carriers changed significantly. The immune system and energy metabolism decreased with development of the disease, while infectious diseases and the metabolism of other amino acids increased with the aggravation of the disease. Cell motility, environmental adaptation, and transcription increased in Group A and decreased with the progression of Hepatitis B, whereas signalling molecules and their interaction, the circulatory system, carbohydrate metabolism, neurodegenerative diseases, metabolism of terpenoids and polyketides, and nucleotide metabolism decreased in Group A and increased with the progression of Hepatitis B.

**Fig. 7:**
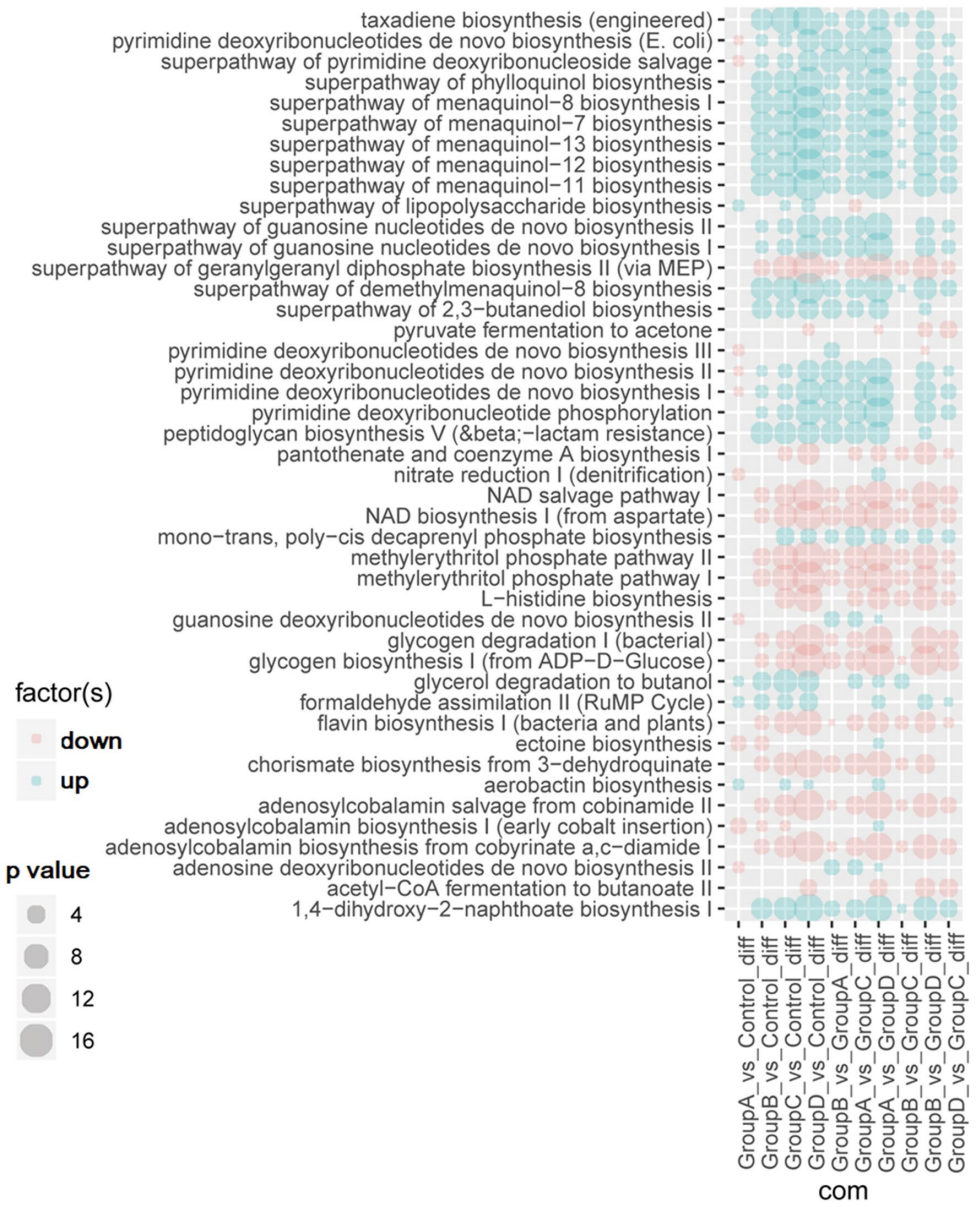
The function of Group A is negatively related to disease progression

Comparison of the significantly different pathways identified using PICRUSt2 was performed between groups and the result is shown in Fig. 7. With the development of the disease, the functional changes of the flora essentially showed the same trend, that is, a certain function was strengthened or weakened. These changes mainly focused on carbohydrate metabolism, nucleotides biosynthesis and menadione biosynthesis. Compared with the other groups, the range and degree of the functional change in Group A was lower; however, the trend of more than 50% was inconsistent (8/14), and most of the changes were functional decline (10/14). One of the 8 inconsistent functional changes was nitrate reduction I (denitrification), while the other seven were related to the biosynthesis of deoxynucleotide. This finding indicates that the predictive function of the intestinal flora in Group A was specific, consistent with the above results regarding the flora composition.

**Fig. 8:**
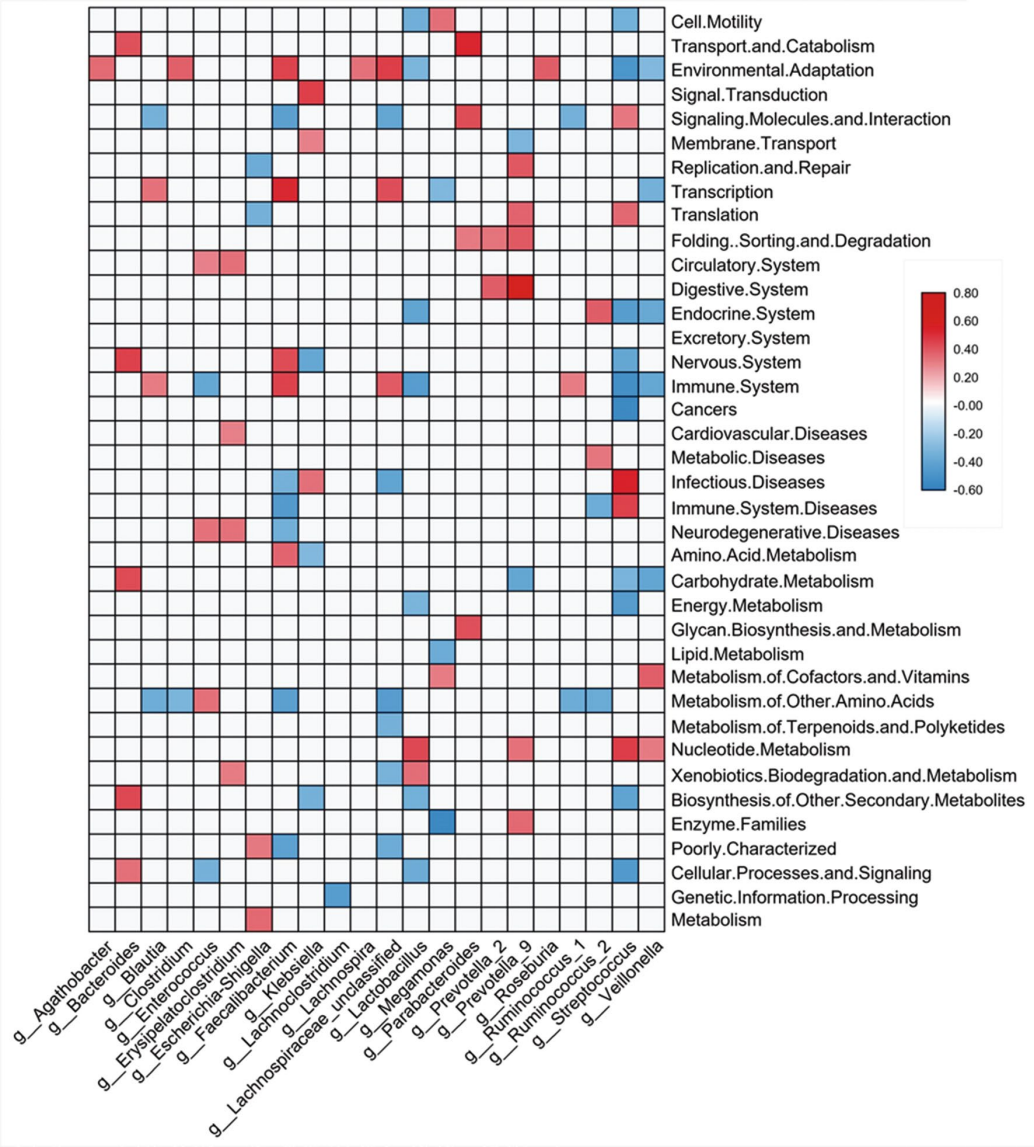
Spearman’s correlation analysis between the 41 predicted biological pathways and the Top 30 genera show that 38 pathways were highly correlated with 22 flora (r > 0.3)

Then, we analysed the relationship between the KEGG pathway and the Top30 flora at the genus level. As many as nine kinds of bacteria showed a high correlation with environmental adaptation (Fig. 8). *G_Agathobacter*, *g_Clostridium*, *g_Faecalibacterium*, *g_Lachnospira*, *g_Lachnospiraceae_unclassified*, and *g_Roseburia* were positively associated with environmental adaptation whereas *g_Lactobacillus*, *g_Streptococcus*, and *g_Veillonella* had a negative relationship with environmental adaptation. The first cluster had advantages in the CK and A groups, while the latter had great advantages in Groups B-D. Therefore, by recognizing the relationship between a strain and the predictive function of environmental adaptation, we could directly determine the role played by the strain. *G_Blautia*, *g_Faecalibacterium*, *g_Lachnospiraceae_unclassified*, and *g_Ruminococcus_1* were positively associated with the immune system, showing a probiotic action, while *g_Enterococcus*, *g_Lactobacillus*, *g_Streptococcus*, and *g_Veillonella* were negatively linked to these functions. In accordance with these findings, *g_Streptococcus* was positively correlated with immune system diseases, while *g_Faecalibacterium* was negatively correlated with immune system diseases. Group A showed decreased biosynthesis of deoxyribonucleotides (Fig. 7), which might be caused by decreased nucleotide metabolism and was positively correlated with *g_Lactobacillus*, *g_Streptococcus,* and *g_Veillonella,* although the amount of *g_Prevotella_9* increased. Similarly, the decline of transport, catabolism and the endocrine system might be caused by changes in related bacteria such as *g_Bacteroides*, *g_Parabacteroides*, *g_Lactobacillus*, *g_Ruminococcus_2*, *g_Streptococcus,* and *g_Veillonella*. In summary, the change in the microbial community may be highly related to the change of the metabolism of the host, which in turn, is related to the development of Hepatitis B.

### Comprehensive exploration of potential biomarkers

In order to intuitively understand the obtained results (Additional files 3, 5, 7, 8, 9, 10, 11, 13, 14: Tables S3, S4, S5, S6, S7, S8, S9, S10, S11), heat map visualization was carried out. Bacteria that were identified by two or more analytical methods are shown in Additional file 15: Figure S4. A total of 47 strains with potential biomarker functions were identified at all levels except for the phylum level. Generally, these strains were abundant. For example, all strains were the Top 3 flora at the phylum level, namely, *Firmicutes*, *Bacteroidetes*, and *Proteobacteria*. According to the comprehensive understanding of the results obtained by various analytical methods, we can understand the influence of various bacteria on the progression of Hepatitis B. For example, *Lactobacillus* is a probiotic [15, 16]. However, in our research, *Lactobacillus* was positively correlated with disease progression, and correction analysis of the predictive function showed that it was negatively correlated with environmental adaptation and the immune system, which indicated that a large amount of *Lactobacillus* accumulation was harmful. Therefore, the relationship between intestinal flora and diseases development is rather complex.

## Discussion

Early prediction of the progression of Hepatitis B is of great value for the diagnosis and treatment of HCC. Therefore, in this study, the features of the gut microbiota in Hepatitis B patients covering four non-cancerous stages, HBV carriers, CHB, liver cirrhosis, and acute-on-chronic liver failure, were investigated using 16S rRNA gene sequencing. We intended to identify the potential role of the gut microbiota in disease progression for early different stages of Hepatitis B patients.

Consistent with previous results, our PCoA results showed that age and sex had no significant effect on the composition of the intestinal flora [9, 10, 17]. Hu and his colleagues showed that the intestinal microbial community was not affected by oral antiviral treatment [9, 18], so antiviral therapy was not regarded as an exclusion criterion in this study. Patients who received antibiotics (including traditional Chinese medicine) within 3 months were excluded because it is reported that some members of the microbial community disappear from the community indefinitely [19]. It is speculated that region and eating habits might affect the flora of Hepatitis B patients [20, 21]. Hence, the participants enrolled in this study were all from the Pearl River Delta in Guangdong Province, South China.

Consistent with previous studies [9, 10, 22], compared with the CK, there was no significant change in the α diversity of HBV carriers or patients with CHB. However, the cohort could be divided into five groups using β diversity, indicating that the intestinal flora has already changed in HBV carriers even though they have no specific symptoms.

The intestinal flora in all reported studies is not completely identical, and the participants come from different regions of China [7, 9, 10]. Therefore, the composition of the intestinal flora may be influenced by diet [20]. With the development of the disease, *g_Roseburia* and *g_Fusobacterium* decreased. *G_Roseburia* produces short-chain fatty acids that could affect colon movement, immune maintenance and anti-inflammatory properties [22]. Therefore, g_roseburia is associated with many metabolic pathways and serves as a beneficial bacteria [23]. In our study, the potential pro-inflammatory strains, g_Escherichia-Shigella, g_Klebsiella *and* g_Enterococcus, increased, similar to previous reports [7, 10]. *G_Lachnospira* [10], *g_Prevotella_9* [24], *g_Clostridium* [7], *g_Eubacterium]_eligens_group,* and *g_Ruminococcus_1* [9, 11] showed a downward trend, while *Veillonella* [9, 10] and g_ysipelatoclostridium were enriched with disease progression. Of note, the first cluster increased and the last two strains decreased in Group A compared with the CK. This phenomenon indicates that the floral composition of HBV carriers has its own particularity compared with those of patients who have clinical symptoms. Similar results were also found according to Gram staining and the microbial function of our cohort. HBV carriers can keep a balance with the virus during their entire life without clinical symptoms. According to our research results, we speculate that the change of the flora of HBV carriers may play an active role in the struggle between the human body and the virus. FMT has been proven to play a potential role in the treatment of refractory diseases including liver disease [5, 6]. Therefore, HBV carriers may be more suitable for FMT than healthy people. Of course, more studies are needed to verify our hypothesis.

In our cohort, 40 strains with an LDA greater than 4 were identified by LEfSe, and these 40 strains belonged to the Top 3 most abundant phylum.

*G_Roseburia* and *g_Faecalibacterium* were the most dominant in the CK, consistent with the previous reports [9, 11]. *G_Prevotella_9*, *g_Clostridium*, *g_Lachnospira*, and *g_Lachnospiraceae_unclassified* were the main bacteria in Group A. With the aggravation of disease, *G_Prevotella_9*, *g_Clostridium*, and *g_Lachnospira* first increased in Group A and then decreased in Groups B-D, which further explained the specificity of Group A. The bacteria *g_Bacteroides* was enriched in patients with CHB, live cirrhosis, and HCC [9], but in our study, it only increased in CHB patients at the order level. The dominant bacteria in Group D were *g_Enterococcus, g_Klebsiella, g_Lactobacillus, g_Veillonella*, and *g_Escherichia*-*Shigella,* which were also in the Top 30 most dominant bacteria in this study. These bacteria have been proved to play potential roles in the development of Hepatitis B [7–10]. This result indicates that there is usually a higher abundance of the flora that may become molecular markers.

Correlation analysis showed that Lactobacillus was positively correlated with disease development, while Clostridium had a negative correlation with disease progression. These findings were confirmed by RDA with the TOP 10 most abundant flora. The serum sodium concentration is considered to be an important predictor of liver transplantation survival [25]. *G_Hungatella* increases in patients with chronic kidney disease and primary IgA nephropathy [26, 27]. In our study, *g_Hungatella* was negatively correlated with the serum sodium concentration. The abundance of *g_Sutterella* decreases in patients with bacteremia or bloodstream infection, indicating that *g_Sutterella* is related to erythrocytopoiesis [28]. In our study, *g_Sutterella* was positively correlated with the red blood cell count. In accordance with this result, the identification of *g_Hungatella* and *g_Sutterella* was not a simple calculation result, but it had practical clinical significance. The Child-Pugh score is widely used for liver function evaluation [29]. We analysed the correlation between the intestinal microflora and the Child-Pugh score and identified 15 highly correlated flora, all of which were discovered by LDA or the AUC. RDA showed that some floras were highly correlated with the staging of Hepatitis B. According to the above results, we can directly judge the role of a bacterium through the relationship between it and the progression of Hepatitis B.

Zeng et al. showed that, compared with the healthy control group, lipid metabolism of patients with Hepatitis B was significantly improved [9]; however, this difference was not found in our study via a PICRUst prediction. The highest metabolic functions were the same as those reported by Zeng and Liu et al*. [*9, 10]. Four of the six pathways had no significant differences, which indicated that these metabolic pathways play an important role in maintaining basic functions. Short-chain fatty acids are the main products of dietary fibre fermentation and reduce mucosal injury and intestinal permeability [30, 31]. In our study, with the development of the disease, Acetyl-CoA fermentation to butanoate II and glycerol degradation to butanol decreased; correspondingly, the immune system and energy metabolism decreased, while infectious diseases increased. Through the detection of microbial community functions, we found that a correlation with the environmental adaptation function could directly identify the role played by a certain bacterium. Bacteria that are positively related to environmental adaptation are beneficial, while those that are negatively related to environmental adaptation are harmful.

We found that some probiotics may play a negative role in disease development. For example, lactic acid bacteria are protobacteria in the gastrointestinal tract and vagina, which are generally considered to be probiotics [32]. In our study, Lactobacillus was negatively associated with environmental adaptation, energy metabolism and the immune system, indicating that it might have negative effects. Although further functional studies are needed to investigate the influence of the increased abundance of g_Lactobacillus on the progression of Hepatitis B, our results indicated that the intake of lactic acid bacteria should be cautious [10]. In summary, through comprehensive analysis of 16S rRNA gene sequencing data, we can basically distinguish the role of bacteria in the progression of Hepatitis B, thus providing data support for FMT.

Although our study provided novel information for the potential role of the intestinal microflora in predicting the progression of Hepatitis B patients in different non-cancerous stages, there are limitations in this study. First, this cross-sectional study only showed the correlation between the gut microflora and the progression of Hepatitis B but did not provide direct causal evidence. Second, the 16S rRNA gene sequencing strategy may not reflect the actual information of the microbial community. Therefore, further studies using metagenomics next-generation sequencing are needed to investigate the potential causal mechanisms that link the gut microbiota and Hepatitis B. Third, although we outlined the significance of intestinal flora based data from asymptomatic HBV carriers, the case number of 24 in this group leads to certain statistical limitations, and further studies with large samples are needed. Additionally, the specificity of the intestinal microflora differences in cases of other liver diseases with different underlying aetiologies in the same population was not under the purview of the present study, which limited the specificity of the data with respect to HBV.

## Conclusions

Consistent with previous studies, our result showed an association between the gut microbiota and Hepatitis B progression. We identified 47 strains with potential biomarker functions at all levels except the phylum level, and we preliminarily analysed their role in the development of Hepatitis B. It should be noted that the floral composition of HBV carriers seems to be more beneficial than that of healthy controls, indicating that they might be suitable donors for FMT.

## Materials and methods

### Participants

Healthy volunteers were regarded as the healthy controls group. Patients treated in the Department of Infectious Diseases of the Third Affiliated Hospital of Sun Yat-Sen University from January 2019 to March 2020 were regarded as candidates. Hepatitis B virus infection was confirmed by being positive for the hepatitis B surface antigen (HBsAg). Hepatitis B was staged according to the Guidelines for the Prevention and Treatment of Chronic Hepatitis B (2015 Edition) issued by the Chinese Medical Association. All the participants in this study had lived in the Pearl River Delta in Guangdong Province for more than 3 years. Patients who were infected by human immunodeficiency virus (HIV) or hepatitis C virus (HCV) or had other diseases, such as alcoholic hepatitis, fatty liver disease, acute or chronic infectious diseases, autoimmune diseases, or non-Hepatitis B liver diseases, were excluded. Patients with a body mass index (BMI) (kg/m2) of less than 18.5 or more than 23.9 were excluded. Patients who with any gastrointestinal disease, which may be linked to a leaky gut and bacterial translocation to the liver, were ruled out. In addition, patients who took antibiotics or traditional Chinese medicine orally within the previous 3 months were excluded. This study was approved by the Ethics Committee of the Third Affiliated Hospital of Sun Yat-Sen University. Written informed consent to participate in this study was provided by the participants.

### Clinical testing

General information such as medical history and residence was collected during the inquiry. Routine bloodwork, reticulocyte counts, liver and kidney function, blood lipid profile, and coagulation function were determined. In the healthy controls group, only routine blood tests, reticulocyte count and liver and kidney function were determined. The patients were tested for virus-related indicators. Liver biopsy or imaging examinations including trans-abdominal ultrasound, computed X-ray tomography (CT), or nuclear magnetic resonance (NMR) were performed when necessary.

### Faecal samples collection and 16S rRNA gene sequencing

Every participant involved in 16S rRNA gene sequencing provided a fresh tail stool sample in the morning. The samples were collected in sterile plastic tubes and stored in a − 80 °C refrigerator as soon as possible. The 16S rRNA sequencing was performed by LC-Bio Technology Co., Ltd, Hang Zhou, Zhejiang Province, China. In brief, DNA was extracted from stool samples using an E.Z.N.A. ^®^Stool DNA Kit (D4015, Omega, Inc., USA) following the manufacturer’s instructions. After determination by 1.2% agarose gel electrophoresis, total DNA was eluted in 50 μL Elution buffer, and stored at − 80 °C until further study.

Regarding sequencing, the 16S rRNA V4 region was amplified using primers 515F(5′-GTGYCAGCMGCCGCGGTAA-3′) and 806R (5′-GGACTACHVGGGTWTCTAAT-3′). The 5′ ends of the primers were tagged with specific barcodes per sample and sequencing universal primers. Polymerase chain reaction products were identified by 2% agarose gel electrophoresis, purified with pure XT beads (Beckman Coulter Genomics Company, Danvers, Massachusetts, USA), and quantified with quantum bits (Nitrogen, USA). After being assessed on an Agilent 2100 Bioanalyser (Agilent, USA) and Library Quantification Kit for Illumina (Kapa Biosciences, Woburn, MA, USA), the libraries were sequenced on NovaSeq PE250 platform according to the manufacturer’s recommendations.

## 16S rRNA data analysis

After sequencing, the raw data were processed as described previously [33–35]. Briefly, FLASH (v1.2.8) was used for paired-end reads merging, and high-quality clean tag filtering was performed according to the fqtrim (v0.94). Chimeric sequences were filtered using Vsearch software (v2.3.4) and DADA2 was used for dereplication. The SILVA classifier (Release 132) is used to normalize feature abundance. Quantitative Insights of Microbial Ecology were used in sequence analysis (QIIIME, version 1.9). The high quality readings with 97% similarity were aggregated into operational taxonomic units (OTUs). Alpha diversity and beta diversity were calculated by OTUs using QIIME and the Mothur program. Differentially abundant bacterial taxa of faecal microbiota that may be considered as biomarkers were identified using the linear discriminant analysis (LDA) effect size (LEfSe) method [36]. The Kruskal–Wallis sum-rank test (α = 0.05) was used to identify features with significantly different abundances among groups, and linear discriminant analysis (LDA) is used to estimate the influence of each feature. A logarithmic LDA score greater than 4 was considered statistically significant. The calculation method of the intestinal dysbiosis index (GDI) was the same as that reported previously  [8].

### Classification analysis and correlation analysis

The Receiver Operating Characteristics (ROC) curve was constructed to investigate the classification efficiency between different groups by using clinical indicators and identified microflora. The pROC package in R is used to build ROC curve, and the AUC threshold was 0.7  [37]. Correlation analysis was conducted to clarify the relationship between clinical indicators and intestinal microflora. According to the specific situation, the threshold value of Pearson coefficient was set to 0.3 or 0.4.

### Potential microbial function prediction

Redundancy analysis (RDA) was performed at each level by using the TOP 10 abundant flora of all samples to clarify the relationship between the intestinal microflora and Hepatitis B staging. Using PICRUst, the corresponding genes with identified characteristics were predicted and mapped to KEGG level -2 and pathway [13]. The obviously different pathways derived from PICRUSt2 among different groups were compared. Correlation analysis was used to study the relationship between prediction function and microbial community at the genus level, with a cut-off value of 0.3.

### Statistical analysis

SPSS 21.0 software (SPSS Inc., Chicago, Illinois) was used for statistical analyses of clinical testing results. Firstly, the data were tested for normality, and data conforming to a normal distribution were expressed as the standard deviation of means. An analysis of variance (ANOVA) was used for comparison between groups. Data that did not conform to a normal distribution were represented by quartiles, and the Mann–Whitney U test was used for comparisons among groups. P < 0.01 was considered statistically significant.

## Supplementary information

**Additional file 1: Table S1.** Clinical information of patients with Hepatitis B in different non-cancerous stages.

**Additional file 2: Table S2.** Discriminatory function built using seven indexes.

**Additional file 3: Table S3.** Comparison results of α diversity for the cohort using the Kruskal–Wallis test.

**Additional file 4: Figure S1.** According to principal component analysis, there were no significant differences in the composition of intestinal flora in terms of gender (a) and age (B).

**Additional file 5: Figure S2.** Abundances of the top top-level microflora at the phylum and genus levels. Accumulation column of flora identified at the phylum level (A) and the Top 30 abundant bacteria at the genus level (B).

**Additional file 6: Table S4.** Abundant bacteria identified at the level of phylum using 16S rRNA gene sequencing.

**Additional file 7: Table S5.** Top 30 abundant bacteria identified at the genus level for each group.

**Additional file 8: Table S6.** Sankey plot of the annotation information, correspondence, and proportion of the two most concerned phyla and genera in the diversity of the flora.

**Additional file 9: Table S7:** Specific bacterial taxonomic group related to Hepatitis B procession revealed by LEfSe. The results show taxa that had an LDA value greater than 3.

**Additional file 10: Table S8.** Specific flora with potential taxonomic function (AUC > 0.7) revealed by the ROC curve.

**Additional file 11: Table S9.** Microflora that were highly correlated with clinical indicators.

**Additional file 12: Figure S3.** Predicted results of the organism-level coverage of functional pathways and biologically interpretable phenotypes using BugBase. Aerobic stats, Contains Mobile Elements stats, Facultatively Anaerobic stats, and Stress Tolerant stats generally changed with the disease progression. Group A showed specificity in the compositions of Gram-positive and Gram-negative flora.

**Additional file 13: Table S10.** Microflora that were highly correlated with the Child–Pugh score stage.

**Additional file 14: Table S11.** Correlation of the predicted diverse functions and the Top 30 abundant flora at the genus level.

**Additional file 15: Figure S4.** Comprehensive exploitation of potential biomarkers. Bacteria identified by two or more analysis methods that belong to the Top3 rich floras. Bacteria in italics played a predictive role of “harmful”, while other bacteria were predicted to be beneficial microorganisms. Different colours indicate different levels. Red: class; orange, order, yellow, family; cyan, genus; blue, species. The same line represents the same classification or the next level of classification. For the same classification, only the leftmost column is marked and the other columns are ignored.

## Data Availability

The datasets used and/or analysed during the current study are available from the corresponding author on reasonable request.

## Abbreviations

HBV: Hepatitis B virus
CHB: Chronic Hepatitis B
HCC: Hepatocellular carcinoma
FMT: Faecal microbiota transplantation
