# Supplementary material for: Potential role of intestinal microflora in disease progression among patients with different stages of Hepatitis B
Source: Gut Pathog. 2020 Oct 27;12:50. doi: 10.1186/s13099-020-00391-4 (PMC7590496; doi:10.1186/s13099-020-00391-4)
Supplement: Supplementary file 1 — Additional file 1: Table S1. Clinical information of patients with Hepatitis B in different non-cancerous stages. [file 13099_2020_391_MOESM1_ESM.docx]

**Additional Table S1 Clinical information for Hepatitis B patients at different noncancerous stages.**

|  | **Control** | **Group1** | **Group2** | **Group3** | **Group4** | **P** |
| --- | --- | --- | --- | --- | --- | --- |
| Age | 33(23-40) | 35(20-58) | 35(11-60)^con*^ | 50.50(33-85)^con,a,b^ | 43(24-65)^con,a,b,c^ | 0.000 |
| WBC | 6.35(3.21-9.36) | 5.99(3.30-11.23) | 5.46(3.30-10.93) | 3.80(1.03-11.37)^con,a,b^ | 6.79(2.25-19.26)^b,c^ | 0.000 |
| RBC | 5.05(4.25-7.54) | 5.11(4.16-6.30) | 4.71(2.69-6.90)^a*^ | 3.84(1.96-33.60)^con,a,b^ | 3.91(2.06-6.17)^con,a,b^ | 0.000 |
| HGB | 153.00(126.00-167.00) | 149.00(119.00-172.00) | 142.00(93.00-174.00)^con*^ | 113.00(61.00-157.00)^con,a,b^ | 118.00(80.00-161.00)^con,a,b^ | 0.000 |
| Hct | 0.44(0.37-0.50) | 0.44(0.36-0.51) | 0.41(0.26-0.51) | 0.32(0.19-0.46)^con,a,b^ | 0.32(0.22-0.46)^con,a,b^ | 0.000 |
| MCV | 87.45(64.50-93.50) | 88.10(61.00-93.20) | 89.70(60.50-100.50) | 88.10(57.90-116.80) | 86.70(54.10-112.60) | 0.261 |
| MCH | 30.05(20.40-32.30) | 30.20(18.90-31.90) | 30.90(19.30-34.80) | 30.60(18.60-39.30) | 30.90(20.50-39.30) | 0.049 |
| MCHC | 342.50(317.00-355.00) | 339.00(310.00-360.00) | 341.00(319.00-379.00) | 341.50(302.00-393.00) | 358.00(327.00-3555.00)^con,a,b,c^ | 0.000 |
| PLT | 237.50(149.00-345.00) | 211.00(118.00-331.00) | 193.00(76.00-342.00)^con^ | 70.00(18.00-266.00)^con,a,b^ | 109.00(28.00-322.00)^con,a,b,c^ | 0.000 |
| NEUT | (0.55±0.08) | (0.59±0.09) | (0.57±0.09) | (0.62±0.12)^con,b^ | (0.69±0.10)^con,a,b,c^ | 0.000 |
| LYMPH | 0.38(0.17-0.50) | 0.30(0.16-0.54) | 0.30(0.13-8.80)^con*^ | 0.27(0.07-7.40)^con,b*^ | 0.22(0.02-9.80)con,a,b,c* | 0.000 |
| MONO | 5.60(0.05-9.10) | 7.00(0.05-9.80) | 0.14(0.05-9.80)^a*^ | 0.13(0.05-9.90)^a^ | 0.12(0.02-9.30)^con*,a,b,c*^ | 0.000 |
| EO | 2.15(0.01-8.80) | 4.80(0.00-9.00) | 1.70(0.00-9.00)^a^ | 2.50(0.00-9.00)^a*^ | 0.02(0.00-9.00)^con,a,b,c^ | 0.000 |
| BASO | 5.00(0.00-9.00) | 4.00(0.00-9.00) | 3.00(0.00-9.00) | 3.00(0.00-9.00) | 0.00(0.00-9.00)^con,a,b,c^ | 0.000 |
| IG | 0.00(0.00-8.00) | 3.00(0.00-9.00) | 3.00(0.00-9.00) | 3.00(0.00-9.00) | 0.20(0.00-9.00) | 0.442 |
| NEUT# | 3.31(1.40-6.87) | 3.45(1.27-6.02) | 3.14(1.69-7.36) | 2.47(0.55-8.20)^con*,a,b*^ | 4.41(1.52-16.02)^con,a*,b,c^ | 0.000 |
| LYMPH# | 2.15(1.40-3.43) | 1.65(1.02-4.13) | 1.58(0.60-3.55)^con^ | 0.99(0.16-3.23)^con,a,b^ | 1.11(0.06-2.93)^con,a,b^ | 0.000 |
| GLU | 4.95(4.44-5.63) | 4.92(3.98-9.36) | 5.51(3.12-27.48) | 5.72(2.70-30.31) | 5.65(1.37-21.44) | 0.060 |
| BUN | 4.67(2.77-7.10) | 4.87(2.12-5.94) | 3.90(2.05-7.26)^a*^ | 5.08(2.18-32.81)^b^ | 3.96(1.17-18.67)^c^ | 0.000 |
| CREAT | 71.00(41.00-99.00) | 76.00(40.00-118.00) | 69.00(45.00-114.00) | 71.00(42.00-286.00) | 65.00(37.00-324.00) | 0.030 |
| eGFR | 108.29(85.27-138.09) | 105.20(61.63-127.85) | 115.62(69.42-162.06)^a^ | 101.46(14.64-125.57)^con*,a*,b^ | 111.34(18.69-158.67)^b*,c^ | 0.000 |
| UA | 385.00(192.00-562.00) | 402.00(264.00-725.00) | 294.00(112.00-564.00)^a^ | 312.00(98.00-767.00)^a^ | 149.00(53.00-528.00)^con,a,b,c^ | 0.000 |
| CHOL | 4.69(3.08-6.22) | 5.04(2.07-9.83) | 3.90(0.97-7.26)^con*,a^ | 3.61(0.69-7.61)^con,a^ | 2.53(0.53-8.58)^con,a,b,c^ | 0.000 |
| TRIG | 0.96(0.42-2.27) | 0.97(0.51-6.01) | 1.43(0.65-7.39)^con*,a*^ | 0.89(0.18-4.42)^b^ | 0.92(0.19-3.55)^b^ | 0.000 |
| HDLC | 1.37(0.82-1.75) | 1.08(0.62-1.80) | 0.58(0.05-4.41)^con,a^ | 0.71(0.07-2.00)^con,a^ | 0.14(0.06-1.56)^con,a,b,c^ | 0.000 |
| AST | 18.00(10.00-25.00) | 22.50(14.00-40.00) | 351.50(22.00-1761.00)^con,a^ | 58.50(13.00-1125.00)^con,a,b^ | 166.00(26.00-2943.00)^con,a,c^ | 0.000 |
| ALT | 18.00(9.00-32.00) | 23.50(11.00-50.00) | 546.00(18.00-2776.00)^con,a^ | 47.00(6.00-353.00)^con,a,b^ | 182.00(18.00-3675.00)^con,a,c^ | 0.000 |
| SL | 1.00(0.56-1.80) | 0.91(0.52-2.09) | 0.59(0.13-3.90)^con,a^ | 1.30(0.49-3.73)^a,b^ | 0.98(0.17-4.63)^b,c^ | 0.000 |
| K | / | 3.90(3.67-4.34) | 3.81(2.88-7.22) | 3.79(2.99-5.19) | 3.74(2.52-5.54) | 0.402 |
| Na | / | 140(139-142) | 139(128-145) | 138(116-145)^a*^ | 138(122-144) ^a,b^ | 0.002 |
| CO_2_ | / | 23.48±1.77 | 25.20±2.51 | 24.36±2.63 | 24.80±3.12 | 0.202 |
| Ca | / | 2.46±0.18 | 2.36±0.15 | 2.20±0.16^a,b^ | 2.29±0.17 ^a,b*,c*^ | 0.000 |
| IPHOS | / | 1.09(0.85-1.5) | 1.11(0.79-1.95) | 1.05(0.73-1.79) | 1.02(0.62-1.75) | 0.109 |
| CysC | / | 0.69(0.56-1.3) | 0.94(0.68-1.91)^a^ | 1.07(0.67-2.65)^a,b*^ | 1.03(0.56-2.26)^a,b*^ | 0.000 |
| β2-MG | / | 1.72(1.41-3.89) | 2.59(1.49-4.9) | 2.57(1.09-5.32) | 2.91(1.76-6.11) | 0.015 |
| TPROT | / | 74.5(63.4-84.4) | 71.4(56.4-84.2) | 66.6(55.1-92.2) ^a,b^ | 61.3(49.1-84.5) ^a,b^ | 0.000 |
| ALB | / | 46.94±3.00 | 41.41±4.49^a^ | 32.92±6.11^a,b^ | 35.17±5.48 ^a,b,c*^ | 0.000 |
| GLB | / | 27.05(20.3-37.3) | 29.5(19.4-40.5) | 32.6(22.8-51.7) ^a,b^ | 27(12.2-55.8)^c^ | 0.000 |
| A/G | / | 1.7(1.3-2.3) | 1.4(0.7-2.1)^a*^ | 1.0(0.4-1.7) ^a,b^ | 1.25(0.5-3.5)^a,c^ | 0.000 |
| TBILI | / | 8.05(6.0-19.6) | 33.85(3.2-819.0)^a^ | 27.7(4.7-292.3)^a^ | 342.5(7.7-864.7) ^a,b,c^ | 0.000 |
| DBILI | / | 2.15(1.3-7.0) | 17.2(1.0-462.0)^a^ | 10.4(1.8-183.6)^a^ | 173.55(3.6-413) ^a,b,c^ | 0.000 |
| IBLI | / | 6.1(4.2-15.5) | 14.2(2.2-357.0)^a^ | 14.75(2.3-108.7)^a*^ | 148.7(4.1-451.7) ^a,b,c^ | 0.000 |
| GGT | / | 19.00(10.0-57.0) | 127.50(11.0-739.0)^a^ | 52.00(9.0-314.0) ^a,b^ | 89.00(19.0-299.0) ^a,b,c*^ | 0.000 |
| ALP | / | 54(34.0-71) | 103.00(52.00-275.00)^a^ | 104.00(52.00-219.00)^a^ | 116.50(83.00-277.00)^a,c^ | 0.000 |
| TBA | / | 4.00(1.4-17.10) | 99.00(4.20-404.3)^a^ | 49.80(2.60-412.60)^a^ | 236.20(4.60-999.5) ^a,b,c^ | 0.000 |
| CHE | / | 8924.50(5254.0-15927.00) | 6282.00(2811.0-11236)^a*^ | 2983.00(1046.0-11339.0) ^a,b^ | 4179.0(1269.0-10181) ^a,b,c*^ | 0.000 |
| PA | / | 261(192-350) | 100(9.0-266.0)^a^ | 77.5(9.0-315.0)^a^ | 37.5(9.0-197.0) ^a,b,c^ | 0.000 |
| AFU | / | 34.0(23-71.00) | 59.00(21.00-108.00)^a^ | 35.00(16.00-75.00)^b^ | 34.00(4.0-158.0)^b^ | 0.000 |
| PT-sec | / | 13.7(12.5-14.2) | 14.4(11.9-24) | 16.7(12.2-29.6) ^a,b^ | 25.8(12.00-45.4) ^a,b,c^ | 0.000 |
| PT% | / | 91.5(86-107) | 85(36-119) | 64.5(27-114) ^a,b^ | 33(16-131)^a,b,c^ | 0.000 |
| PT-INR | / | 1.06(0.96-1.10) | 1.1(0.91-2.14) | 1.34(0.93-2.7) ^a,b^ | 2.36(0.86-4.72) ^a,b,c^ | 0.000 |
| PT-Ref.t | / | 13.1(12.9-13.4) | 13.4(12.9-13.4)^a^ | 13.4(12.8-13.4)^a*^ | 13.1(12.8-13.4)^b,c^ | 0.000 |
| FIB-l | / | 2.88±0.48 | 2.64±0.59 | 2.16±0.67 ^a,b^ | 1.65±0.69 ^a,b,c^ | 0.000 |
| FIB-sec | / | 17.5(14.2-22.6) | 18.85(12.2-31.8) | 20.9(12.4-33.6) | 21.9(11.6-33.6) | 0.045 |
| APPR | / | 1.07(0.85-1.24) | 1.19(0.95-1.55)^a*^ | 1.22(0.96-1.85)^a^ | 1.5(1.00-3.01) ^a,b,c^ | 0.000 |
| TT | / | 16.75±0.97 | 18.53±1.31^a*^ | 18.51±3.24^a*^ | 20.99±2.20 ^a,b,c^ | 0.000 |
| TT-R | / | 0.96(0.86-1.05) | 1.05(0.91-1.32)^a^ | 1.06(0.01-1.41)^a^ | 1.2(0.91-1.58) ^a,b,c^ | 0.000 |

Data were first tested for normality, and data that met normal distribution were expressed as means ± SD. Paired sample t test was used for comparisons between groups. Data that did not meet normal distribution were indicated as quartiles, and the Mann-Whitney U test was used for group comparisons.

Note: con/con*, p<0.01/0.01<p<0.05 while compared with Control; a/a*, p<0.01/0.01<p<0.05 while compared with Group A; b/b*, p<0.01/0.01<p<0.05 while compared with Group B; c/c*, p<0.01/0.01<p<0.05 while compared with Group C; WBC, white blood cell; RBC, red blood cell; HGB, hemoglobin; Hct, hematocrit; MCV, mean corpuscular volume; MCH, mean corpuscular hemoglobin; MCHC, mean corpuscular hemoglobin concentration; PLT, platelet; NEUT, neutrophils; LYMPH, lymphocytes; MONO, monocyte; EO, eosinophils; BASO, basophils; IG, immature granulocyte; NEUT#, neutrophils absolute value; LYMPH#, lymphocytes absolute value; MONO#, monocyte absolute value; EO#, eosinophils absolute value; GLU, glucose; BUN, blood urea nitrogen; CREAT, creatinine; eGFR, estimated glomerular filtration rate; UA, uric acid; CHOL, total cholesterol; TRIG, triglyceride; HDLC, high density lipoprotein cholesterol; AST, aspartate aminotransferase; ALT, alanine aminotransferase; S/L, aspartate aminotransferase/alanine aminotransferase; K, potassium; Na, sodium; Cl, chlorine; CO_2_, carbon dioxide; Ca, calcium; IPHOS, inorganic phosphorus; CysC, cystatin c; β2-MG, macroglobulin; TPROT, Total protein; ALB, albumin; GLB, globulin; A/G, albumin/globulin; TBILI, total bilirubin; DBILI, direct bilirubin; IBILI, indirect bilirubin; GGT, glutamyl transpeptidase; ALP, alkaline phosphatase; TBA, total bile acid; CHE, choline esterase; PA, prealbumin; AFU, fucosidase; PT-sec, prothrombin time; PT%, Prothrombin activity%; PT-INR, prothrombin time-international normalized ratio; PT-Ref.t, prothrombin reference time; FIB-l, fibrinogen level; FIB-sec, fibrinogen; APTT, activated partial thromboplastin time; APPR, activated partial prothrombin ratio; TT, thrombin time; TT-R, thrombin ratio.
