# Supplementary material for: Potential role of intestinal microflora in disease progression among patients with different stages of Hepatitis B
Source: Gut Pathog. 2020 Oct 27;12:50. doi: 10.1186/s13099-020-00391-4 (PMC7590496; doi:10.1186/s13099-020-00391-4)
Supplement: Supplementary file 2 — Additional file 2: Table S2. Discriminatory function built using seven indexes. [file 13099_2020_391_MOESM2_ESM.docx]

**Additional Table S2 Discriminatory function built using clinical indicators.**

|  | Group A | Group B | Group C | Group D |
| --- | --- | --- | --- | --- |
| Platelet | .013 | .018 | -.012 | .010 |
| Total protein | .015 | -.094 | .088 | -.081 |
| Albumin | 2.887 | 3.205 | 2.615 | 3.104 |
| Total bilirubin | .023 | .027 | .022 | .038 |
| Prealbumin | -.081 | -.173 | -.125 | -.143 |
| Prothrombin activity% | .718 | .882 | .741 | .711 |
| Prothrombin time | 11.258 | 11.999 | 10.962 | 12.005 |
| Constant | -189.343 | -206.086 | -167.177 | -196.624 |
