# Supplementary figures and images for: Potential role of intestinal microflora in disease progression among patients with different stages of Hepatitis B
Source: Gut Pathog. 2020 Oct 27;12:50. doi: 10.1186/s13099-020-00391-4 (PMC7590496; doi:10.1186/s13099-020-00391-4)

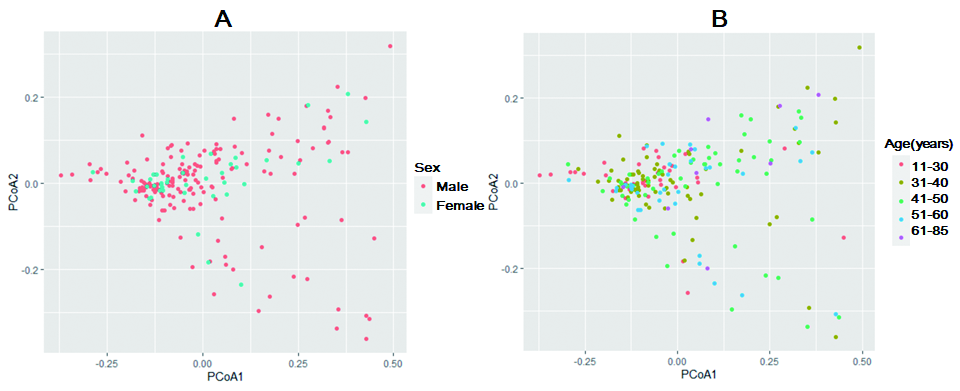

Supplement: Supplementary file 4 — Additional file 4: Figure S1. According to principal component analysis, there were no significant differences in the composition of intestinal flora in terms of gender (a) and age (B). [file 13099_2020_391_MOESM4_ESM.tif]

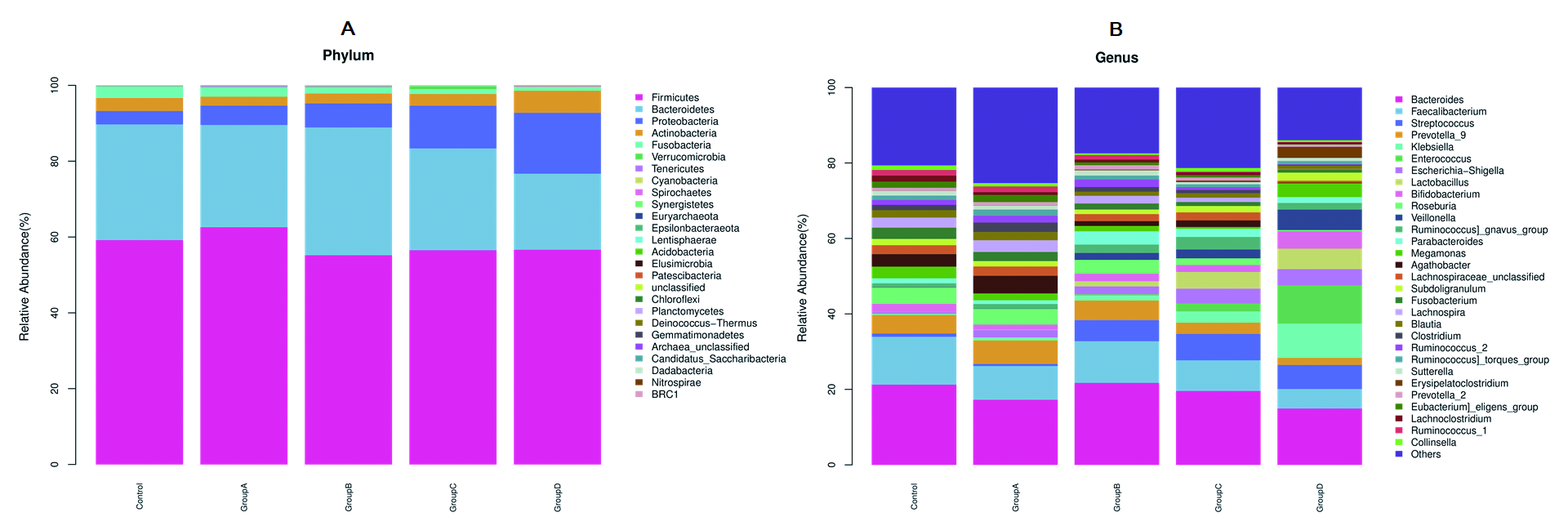

Supplement: Supplementary file 5 — Additional file 5: Figure S2. Abundances of the top top-level microflora at the phylum and genus levels. Accumulation column of flora identified at the phylum level (A) and the Top 30 abundant bacteria at the genus level (B). [file 13099_2020_391_MOESM5_ESM.tif]

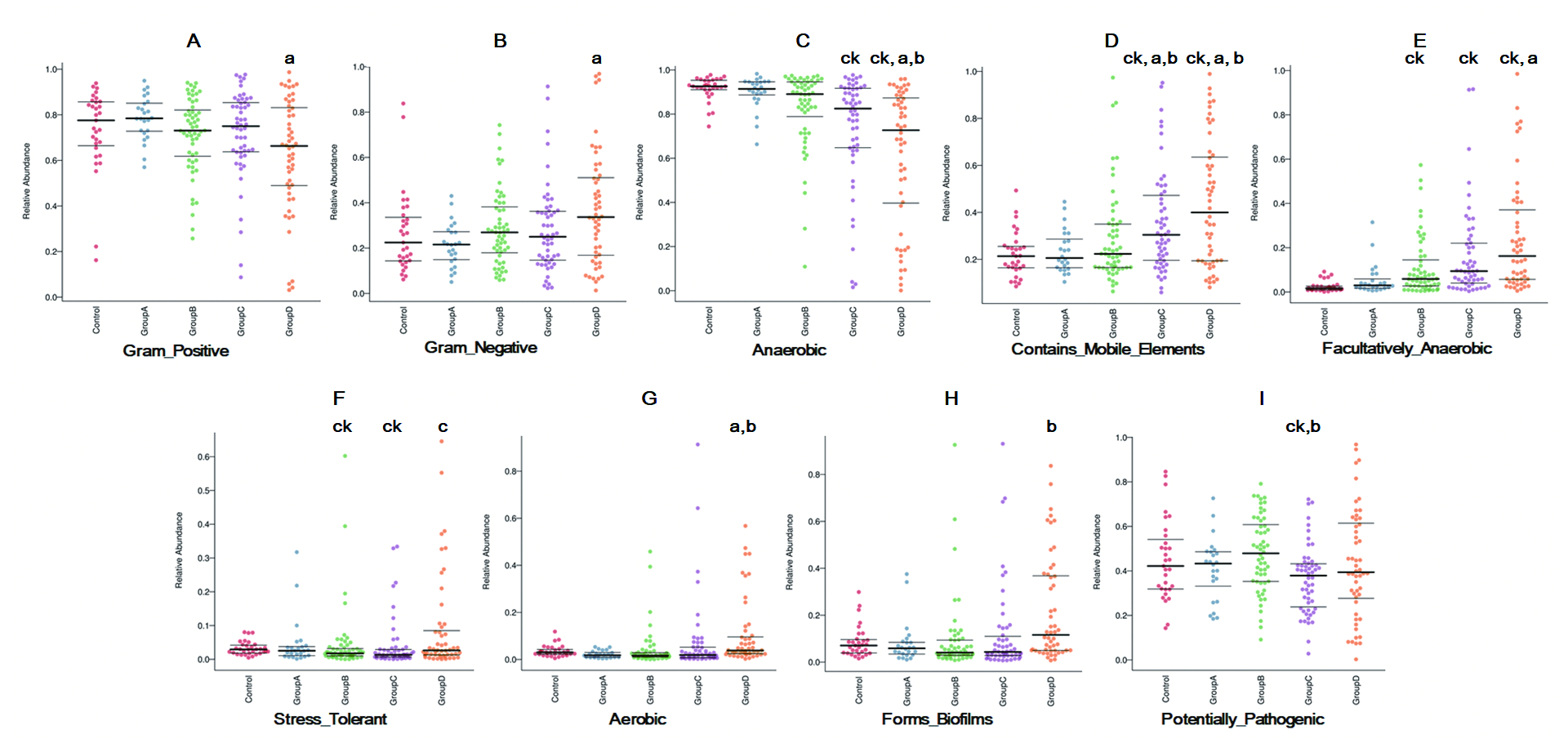

Supplement: Supplementary file 12 — Additional file 12: Figure S3. Predicted results of the organism-level coverage of functional pathways and biologically interpretable phenotypes using BugBase. Aerobic stats, Contains Mobile Elements stats, Facultatively Anaerobic stats, and Stress Tolerant stats generally changed with the disease progression. Group A showed specificity in the compositions of Gram-positive and Gram-negative flora. [file 13099_2020_391_MOESM12_ESM.tif]

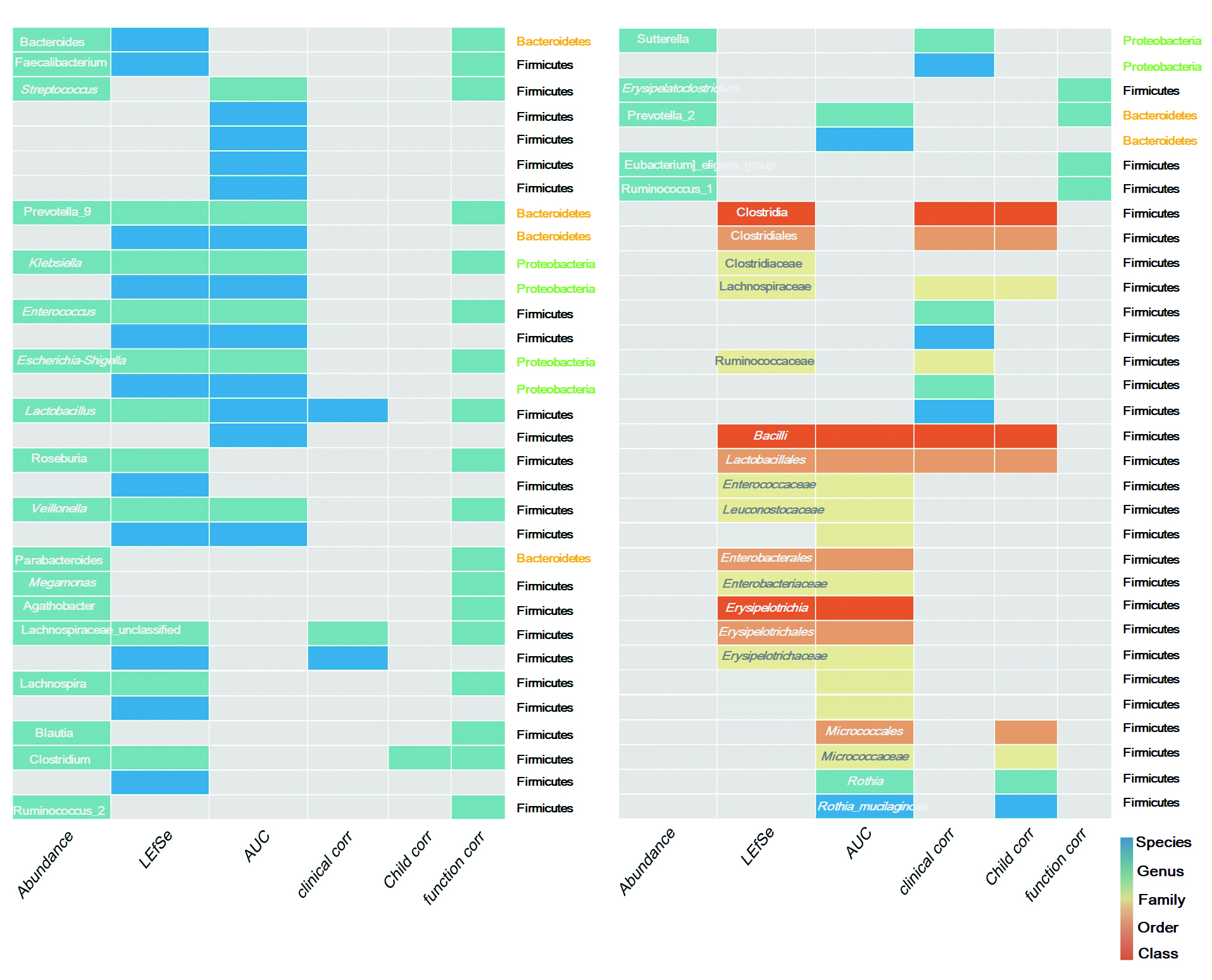

Supplement: Supplementary file 15 — Additional file 15: Figure S4. Comprehensive exploitation of potential biomarkers. Bacteria identified by two or more analysis methods that belong to the Top3 rich floras. Bacteria in italics played a predictive role of “harmful”, while other bacteria were predicted to be beneficial microorganisms. Different colours indicate different levels. Red: class; orange, order, yellow, family; cyan, genus; blue, species. The same line represents the same classification or the next level of classification. For the same classification, only the leftmost column is marked and the other columns are ignored. [file 13099_2020_391_MOESM15_ESM.jpg]
